# Investigation of Chemical Profiles of Different Parts of *Morus alba* Using a Combination of Molecular Networking Methods with Mass Spectral Data from Two Ionization Modes of LC/MS

**DOI:** 10.3390/plants10081711

**Published:** 2021-08-19

**Authors:** Seong Yeon Choi, Jinyoung Park, Juyeol Kim, Jiho Lee, Heejung Yang

**Affiliations:** Laboratory of Natural Products Chemistry, College of Pharmacy, Kangwon National University, Chuncheon 24341, Korea; sych426@kangwon.ac.kr (S.Y.C.); zzzzinoooo@gmail.com (J.P.); kkyedcrt@gmail.com (J.K.); jiho3232@kangwon.ac.kr (J.L.)

**Keywords:** Moraceae, *Morus alba*, GNPS, molecular networking, merge polarity networks

## Abstract

Plants produce numerous secondary metabolites with diverse physicochemical properties. Because different parts of a single plant produce various components, several spectroscopic methods are necessary to inspect their chemical profiles. Mass spectral data are recognized as one of the most useful tools for analyzing components with a wide range of polarities. However, interpreting mass spectral data generated from positive and negative ionization modes is a challenging task because of the diverse chemical profiles of secondary metabolites. Herein, we combine and analyze mass spectral data generated in two ionization modes to detect as many metabolites as possible using the molecular networking approach. We selected different parts of a single plant, *Morus alba* (Moraceae), which are used in the functional food and medicinal herb industries. The mass spectral data generated from two ionization modes were combined and analyzed using various molecular networking workflows. We confirmed that our approach could be applied to simultaneously analyze the different types of secondary metabolites with different physicochemical properties.

## 1. Introduction

Medicinal plants have been long-time sources of therapeutic agents; throughout the years, they have provided directions for discovering new chemical entities for new medicine [1,2]. However, the wide range of components in a single medicinal herb hinders the interpretation of the biological impact of the extract or the isolation of novel secondary compounds. Plants produce and accumulate different primary and secondary metabolites, which are mediated by their biosynthetic pathways when adapting to different environmental conditions (such as temperature, rainfall, soil, diseases, and herbivores) [3,4,5]. Additionally, the different plant parts, including bark, twigs, leaves, flowers, and fruits, produce unique metabolites that are used as therapeutic sources in oriental medicines [6,7,8].

High-resolution mass spectrometry (HRMS) is a powerful technique for analyzing many metabolites in a short time. It uses state-of-the-art ultra-high-performance liquid chromatography and ionizers and is being widely utilized in the field of natural product research [9,10]. Because many metabolites in natural products have various physicochemical properties, two different types of mass spectral data obtained under positive and negative ionization modes are usually measured for a sample, which are then compared with each other data when studying their chemical profiles [11,12,13]. Recently, the Global Natural Product Social Molecular Networking (GNPS) platform has been regarded by natural product chemists as an alternative method for processing huge HRMS data generated from the secondary metabolites of natural products [14]. This platform enables the simultaneous annotation of numerous metabolites from natural product extracts by comparing the annotations from mass spectral databases and from in silico methods [15,16].

Recently, the workflow called Merge Polarity Networks was introduced, which allowed the mass spectral data from two ion types to be merged based on retention times and *m/z* values. This workflow enabled the visualization and exploration of molecular networks generated from positive and negative ion modes into a single network, which can be either classical molecular networking (MN) or feature-based MN (FBMN) [17] tasks. In the merged network, when both nodes in the positive and negative networks are annotated as the same molecule by the tanimoto scoring between two molecules, two nodes are connected to each other.

*Morus alba* (Moraceae, *M. alba*) is cultivated worldwide. All parts of this widely grown plant, including fruits, twigs, leaves, and bark, have been used as medicines or food ingredients in East Asian countries, such as Korea, Japan, and China for different purposes [18]. The fruits of *M. alba*, commonly known as white mulberry, nourish the blood, treat fatigue and anemia, and are widely made into juices for refreshment. In addition, the bark and leaves of *M. alba* have a wide range of biological applications, including for use as a blood pressure depressant and as cathartic, analgesic, diuretic, antitussive, sedative, hypotensive, and edema treatment medicine [18,19,20]. The constituents of *M. alba* have been intensively studied. This plant is a rich source of flavonoids, saponins, and other minor components, such as tannins, coumarins, alkaloids, and organic acids, but they are differentially distributed in various parts of the plant [18].

In this study, we attempted to inspect the chemical profiles of the fruits, twigs, leaves, and bark of *M. alba* as well as the metabolic changes between the parts, using a combination of MN, Network Annotation Propagation (NAP), MolNetEnhancer, and Merge Polarity Networks compared to other chemical profiling studies of *M. alba* samples [21,22,23,24]. We confirmed that the molecular changes between samples were more easily detected and analyzed through these approaches.

## 2. Results and Discussion

**Figure 1:**
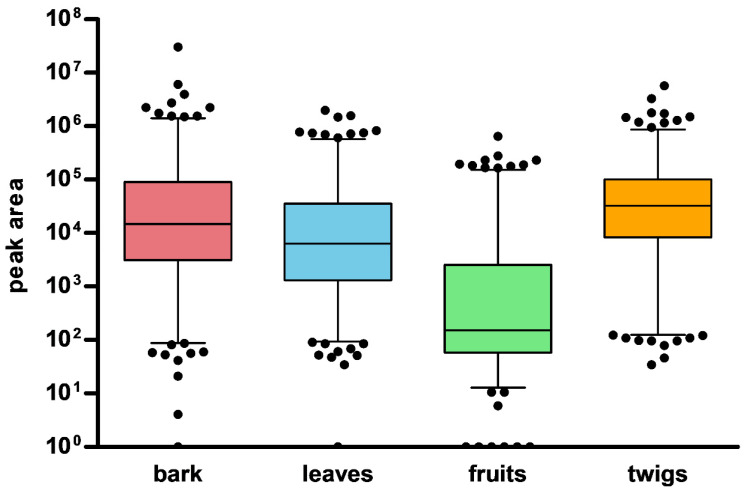
Distribution pattern of nodes in different parts of *M. alba*.

The 427 nodes were generated from the positive mode, whereas 189 nodes were generated from the negative mode. Additionally, the different parts showed different patterns of the nodes corresponding to the peaks in the chromatograms (Figure 1). The peaks from the fruits had significantly lower intensities in both ionization modes compared to the other parts. Next, we inspected the distribution of the nodes from each part using a combination of MN methods.

**Figure 2:**
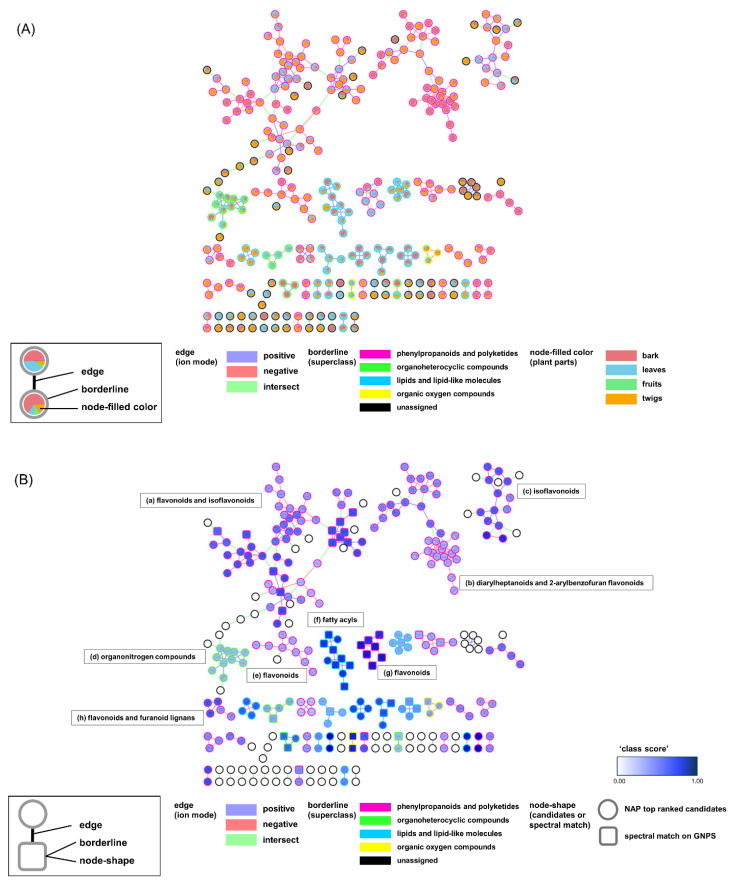
(**A**) Molecular networking (MN) of the distribution of plant parts (bark, leaves, fruits, and twigs) based on the peak area of the MS1 ion peak. (**B**) MN of the annotated structure based on Network Annotation Propagation (NAP). Molecular families (a)–(h) are highlighted in (**B**). Square node: nodes in the spectral library; round nodes: nodes annotated by NAP.

A total of two MNs were generated by combining the original MN [17], NAP [16], and MolNetEnhancer [15] in the GNPS platform (Figures S1–S3, Supplementary Materials). Each network in the positive and negative modes had 90 and 284 single nodes, respectively. These nodes were not connected to other nodes and were not recognized by either NAP or MolNetEnhancer. Next, we merged the two networks from the two ionization modes using the Merge Polarity Networks function in the GNPS platform. In the merged network, the single nodes were reduced to 313 from the original 374 in the two networks. A total of Sixty-one single nodes were connected to each other and formed clusters (Figure 2). A total of 303 nodes that had connections with other nodes remained in the molecular network.

The attributes of the nodes and edges provided structural information on the chemical profiles. The pie chart in Figure 2A represents the ion intensities corresponding to the peaks in the liquid chromatography (LC)/MS spectra from the different plant parts. The nodes from the same part were gathered in sub-clusters. Interestingly, many nodes from the twigs were intermediately shared with those from the stems and leaves. This implies that the twigs act as tissues connecting the stems and leaves in the middle. Edge-connecting nodes from negative and positive ion modes are displayed in green. They generate larger clusters compared to those in the networks from each ion mode (Figures S1 and S2, Supplementary Materials). The borders of the nodes display the chemical superclasses of the nodes, as annotated by MolNetEnhancer [15]. Among the total 303 nodes that were annotated, 169 were “phenylpropanoids and polyketides,” 19 were “organoheterocyclic compounds,” 44 were “lipids and lipid-like molecules,” and 5 were “organic oxygen compounds.” MN was re-drawn for an in-depth inspection of the chemical profiles using the NAP annotation tool [16] (Figure 2B). The chemical classes for the clusters had more specific annotations. Flavonoid-related structures, such as flavonoids, isoflavonoids, and 2-arylbenzofuran flavonoids, were extensively annotated in the entire network (Table 1).

**Figure 3:**
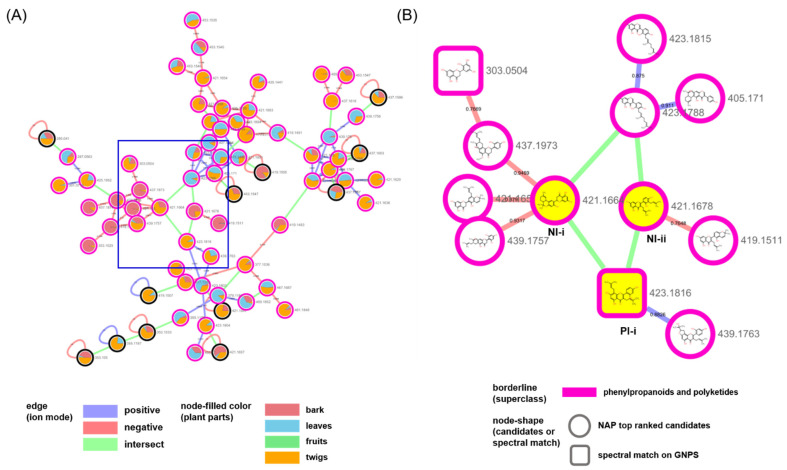
(**A**) Molecular network of the biggest cluster annotated as “flavonoids and isoflavonoids” in Figure 2. (**B**) NAP results of the nodes of the blue rectangle in (**A**); NI-i (annotated as dicyclokuwanon EA), NI-ii (annotated as kuwanon T) and PI-i (connected to NI-i and NI-ii).

**Figure 4:**
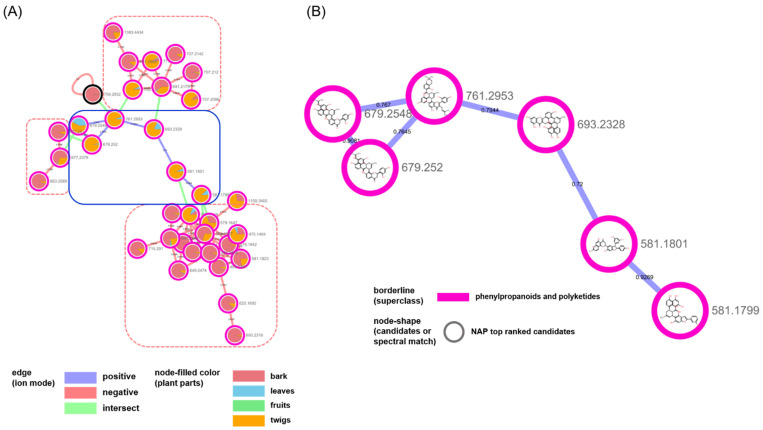
(**A**) Molecular network of the cluster annotated as “diarylheptanoids and 2-arylbenzofuran flavonoids” in Figure 2. (**B**) NAP results of nodes of the blue rounded rectangle in (**A**).

We examined the largest cluster in the network in detail to evaluate the efficiency of our approach in the present study (Figure 3 and Figure 4). The nodes that were created by different ion modes were grouped based on the structures annotated as “flavonoids and isoflavonoids” (Figure 3A,B). The PI-i node generated in the positive ion mode was kuwanon C [25], which was confirmed not only as a match in the GNPS spectral library but also as a prenylated flavonoid and a marker compound in *M.alba* (Figure 3B). The PI-i was connected to two nodes, NI-i (annotated as dicyclokuwanon EA) [26] and NI-ii (annotated as kuwanon T) [27], from the negative ion mode. The nodes from the two ion modes were connected by the *m/z* values of their precursor ions and their retention times. Branched nodes that were linked with these yellow nodes showed other prenylated flavonoid-type structures.

The nodes in the second biggest cluster mainly originated from bark and twigs, (Figure 4). A total of three clusters, which were classified as “diarylheptanoids” and “2-arylbenzofuran flavonoids” in the negative mode (red-dotted rectangle with rounded corners), were annotated as the derivatives of “Diels–Alder-type flavonoids“, which were classified in the positive mode (blue rectangle with rounded corners) and were isolated from *M. alba* and biosynthesized by the enzymatic reaction between dehydroprenyl diene or arylbenzofuran derivatives and flavonoids ([28,29]. The combination of in silico annotation methods in the GNPS platform helped us investigate the metabolic profiles of the plant. We could easily inspect the distribution or changes of the metabolites in different plant parts of *M. alba*. With the exception of the chemical types related to flavonoids, the clusters for “organonitrogen compounds” and “fatty acyls” mainly originated from the metabolites in the fruit and leaf samples, respectively (Figures S4 and S5, Supplementary Materials). Additionally, the minor groups that were characterized by either prenylation at C-6 (Figure S6, Supplementary Materials) or as glycosylated flavonoids (Figure S7, Supplementary Materials) were also annotated. Our approach was successful for the known compounds reported in *M. alba;* however, clusters with irregular structures were also grouped. For example, the small cluster consisting of five nodes indicated a highly-prenylated biflavonoid from the two ion modes that do not exist in nature (Figure S8, Supplementary Materials).

Because a single natural product produces tons of metabolites, the chemical profiling of a sample is a challenging step for the inspection of the metabolic changes and the discovery of druggable candidates in natural products. MN approaches are continuously being developed and applied to research on natural products. There are still limitations for the exact identification of the MS spectral peaks, and so efforts are constantly being made to reduce the process and to increase the reliability of the MN results. In this study, our approaches also suggested that several in silico methods based on the MN may be one of the complementary methods for the chemical profiling of natural products with high reliability.

## 3. Materials and Methods

### 3.1. Plant Material

The bark, leaves, fruits, and twigs of *M. alba* were purchased from an online herbal medicine market (http://hanyakjae.net (accessed on 19 March 2019)) in March 2019. These plants were collected in Korea, and the cultivation sites were Cheorwon for the bark, Yeongcheon for the leaves and twigs, and Sangju for the fruits.

### 3.2. Sample Preparation

More than 3 g of each sample was extracted with 100% ethanol (repeated three times with 3 h of sonication) and were concentrated in vacuo. The extract was diluted with 100% methanol to a concentration of 10 mg/mL. The collected solution was filtered through a regenerated cellulose (RC) membrane filter for LC-MS/MS analysis.

### 3.3. LC-MS/MS Analysis Condition

LC-MS/MS analyses were performed on a Waters Xevo G2 qTOF mass spectrometer (Waters MS Technologies, Manchester, UK) with a UPLC system using electrospray ionization (ESI). Chromatographic separations were performed on a Waters Acquity BEH C18 (100 × 2.1 mm^2^, 1.7 μm) column. The mobile phase consisted of H_2_O (A) and acetonitrile (B), which were both acidified with 0.1% formic acid and were eluted in gradient mode as follows: 5–95% B (0–20 min), 95–100% B (20–20.1 min), 100% B (20.1–22 min), 100–5% B (22–22.1 min), and 5% B (22.1–24 min). The sample organizer and column temperatures were maintained at 20 °C and 40 °C, respectively. The sample (1.0 μL injected) analyses were performed in an optimized FastDDA mode [30], starting with a full MS survey scan with *m/z* values between 100 and 1200 Da followed by MS/MS scans for the three most intense ions (scan time: 100 ms). The ESI conditions were set at a 1.5 kV capillary voltage, a 35 V cone voltage, a 120 °C source temperature, a 350 °C desolvation temperature, a 50 L/h cone gas flow, and a 800 L/h desolvation gas flow. High purity nitrogen and argon gases were used as the nebulizer and auxiliary gases, respectively.

### 3.4. LC-MS/MS Data Analysis

#### 3.4.1. Molecular Networking

The aligned results of the MS1 and MS/MS data of each sample were converted to an mgf format using the MZmine software (Ver. 2.4.2) [31]. The MN was constructed through the GNPS website (https://gnps.ucsd.edu (accessed on 8 July 2020)) [9]. The parent mass tolerance and the MS/MS fragment ion tolerance were set to 0.02 Da. The MN was created using a minimum cosine score above 0.7 and more than four minimum-matched fragment ions and peaks. The MS/MS spectra were filtered by selecting only the top six peaks in the ±50 Da window across each spectrum. The mass spectral data in the MN were then searched in the GNPS spectral libraries. The MN was visualized with Cytoscape 3.8.2 (http://www.cytoscape.org/ (accessed on 8 July 2020)) [32]. Information on the MN nodes and edges was found in the GNPS repository as follows:

PI mode; task ID = 1f2c11689cf94d1b9fbf04d299daf72f

NI mode; task ID = dc4c5c95ce224f3bb6f78193009afb9c

#### 3.4.2. In Silico Annotation Tool, Network Annotation Propagation (NAP)

The MN was used to confirm the propagation of in silico annotation with Network Annotation Propagation (NAP) (https://proteomics2.ucsd.edu/ProteoSAFe/?params=%7B%22workflow%22:%22NAP_CCMS2%22%7D (accessed on 8 July 2020)) [16] using the following parameters: the 10 first candidates were used for consensus score and 10 ppm accuracy for the exact mass candidate search. Finally, we used the GNPS, DNP, SUPNAT, and DRUGBANK structural databases.

#### 3.4.3. Chemical Class-Level Annotation Tool, MolNetEnhancer

MolNetEnhancer is a workflow that combines and outputs molecular networks with other tools such as NAP, DEREPLICATOR, VARQUEST, and MS2LDA. (https://gnps.ucsd.edu/ProteoSAFe/index.jsp?params=%7B%22workflow%22:%22MOLNETENHANCER%22%7D (accessed on 12 July 2020)) [15]. The GNPS MN task ID is essential, and an optional input of the task ID to be integrated is possible.

#### 3.4.4. Merge Polarity Networks

The Merge Polarity Networks workflow (https://ccms-ucsd.github.io/GNPSDocumentation/mergepolarity/ (accessed on 8 July 2020)) is a tool that combines MN results in different ion modes to PI and NI modes. Both classical and feature-based molecular networking (FBMN) jobs are available, and each task requires an ID. The retention time tolerance for aligning masses between the two tasks must be specified in minutes for the FBMN tasks and in seconds for the classical molecular networking tasks. The ppm tolerance must also be specified for merging. In our study, a ppm tolerance of 50 and a RT tolerance of 10 s were used.

## 4. Conclusions

Because a large amount of MS data often exceeds the limits of manual inspection, the digitization of raw MS data is a useful method for researchers to access and translate the complexity of specialized metabolites in natural products. MN has been introduced and has become a powerful technique in the natural products research field. The combination of in silico annotation tools and MN can help in inspecting changes in the chemical profiles of multiple samples. In the present study, thousands of metabolites from different parts of *M. alba* were classified into several classes by the scaffolds. The metabolites produced in this plant, such as prenylated flavonoids and isoflavonoids, were visualized based on structural similarities and were successfully annotated by two annotation tools. Additionally, we combined MS data from two ion modes to improve the annotation rate of the nodes in the MN. As a result, the clusters that were separated in each ion mode were connected to each other by comparing the retention times and *m/z* values of the ion peaks. The combined MN reliably annotates nodes; moreover, it proposes the chemical class of unassigned nodes. Our approach is expected to reliably facilitate a simple inspection of chemical profiles from a plethora of metabolites in natural products.

## Figures and Tables

**Table 1:** Summary of the chemical classes of the structures annotated from NAP.

**positive ion mode**	**bark (%)**	**leaves (%)**	**fruits (%)**	**twigs (%)**
fatty acyls	55.02	36.17	77.64	14.00
flavonoids	9.70	48.58	1.96	77.06
isoflavonoids	1.27	11.62	0.29	7.54
organonitrogen compounds	2.33	2.48	11.01	0.95
peptidomimetics	0.88	0.00	2.44	0.24
prenol lipids	5.69	0.72	3.85	0.20
tetrapyrroles and derivatives	25.11	0.43	2.82	0.00
sum (%)	100.00	100.00	100.00	100.00
**negative ion mode**	**bark (%)**	**leaves (%)**	**fruits (%)**	**twigs (%)**
2-arylbenzofuran flavonoids	9.43	0.82	1.13	5.26
anthracenes	1.27	0.01	0.02	4.55
benzopyrans	48.71	1.36	0.06	4.20
carboxylic acids and derivatives	0.26	0.02	0.01	0.18
coumarins and derivatives	1.20	1.80	5.58	3.81
diarylheptanoids	8.91	2.08	0.12	13.78
fatty acyls	1.99	15.48	23.77	5.40
flavonoids	19.01	44.46	29.37	42.78
furanoid lignans	6.04	0.38	0.04	6.55
isoflavonoids	2.83	3.47	0.24	8.22
organooxygen compounds	0.29	25.69	30.76	4.21
prenol lipids	0.04	0.64	0.41	1.02
steroids and steroid derivatives	0.00	3.81	8.50	0.05
sum (%)	100.00	100.00	100.00	100.00

## Data Availability

The data presented in this study are available upon request from the corresponding author.

## Supplementary Materials

The following are available online at https://www.mdpi.com/article/10.3390/plants10081711/s1, Figure S1: The original MN results of (A) positive and (B) negative ion mode, Figure S2: The NAP results of (A) positive and (B) negative ion mode, Figure S3: The MolNetEnhancer results of (A) positive and (B) negative ion mode, Figure S4: (A) The molecular network and (B) NAP results of the cluster annotated as ‘organonitrogen compounds’, Figure S5: (A) The molecular network and (B) NAP results of the cluster annotated as ‘fatty acyls’, Figure S6: (A) The NAP results of the cluster annotated as ‘isoflavonoids’. (B) Highlighted results of nodes of the blue rectangle in (A) and characterized by prenylation at 6-carbon, Figure S7: (A) The molecular network and (B) NAP results of the cluster annotated as ‘flavonoids’, Figure S8: (A) The molecular network and (B) NAP results of the cluster annotated as ‘flavonoids and furanoid lignans’.

## References

[1] Koehn F.E., Carter G.T. (2005). The evolving role of natural products in drug discovery. Nat. Rev. Drug Discov..

[2] Harvey A.L., Edrada-Ebel R., Quinn R.J. (2015). The re-emergence of natural products for drug discovery in the genomics era. Nat. Rev. Drug Discov..

[3] Richard N.B., Roger M. (1994). Wallsgrove Tansley Review No. 72. Secondary Metabolites in Plant Defence Mechanisms. New Phytol..

[4] Gutbrodt B., Gutbrodt B., Dorn S., Dorn S., Mody K., Mody K. (2012). Drought stress affects constitutive but not induced herbivore resistance in apple plants. Arthropod-Plant. Interact..

[5] Sampaio B.L., Edrada-Ebel R., Da Costa F.B. (2016). Effect of the environment on the secondary metabolic profile of Tithonia diversifolia: A model for environmental metabolomics of plants. Sci. Rep..

[6] Rai A., Saito K., Yamazaki M. (2017). Integrated omics analysis of specialized metabolism in medicinal plants. Plant J..

[7] Wangchuk P. (2018). Therapeutic Applications of Natural Products in Herbal Medicines, Biodiscovery Programs, and Biomedicine. J. Biol. Act. Prod. Nat..

[8] Gosetti F., Chiuminatto U., Martinotti S., Bolfi B., Ranzato E., Manfredi M., Marengo E. (2016). Characterization of the Volatile and Nonvolatile Fractions of Heartwood Aqueous Extract from Pterocarpus marsupium and Evaluation of Its Cytotoxicity against Cancer Cell Lines. Planta Med..

[9] Wang M., Carver J.J., Phelan V., Sanchez L., Garg N., Peng Y., Nguyen D.D., Watrous J., Kapono C.A., Luzzatto-Knaan T. (2016). Sharing and community curation of mass spectrometry data with Global Natural Products Social Molecular Networking. Nat. Biotechnol..

[10] Wolfender J., Litaudon M., Touboul D., Queiroz E.F. (2019). Innovative omics-based approaches for prioritisation and targeted isolation of natural products—New strategies for drug discovery. Nat. Prod. Rep..

[11] Rockwood A.L., Kushnir M.M., Clarke N.J. (2018). Mass Spectrometry. Princ. Appl. Clin. Mass Spectrom..

[12] Charles L., Laure C., Lutz J., Roy R.K. (2016). Tandem mass spectrometry sequencing in the negative ion mode to read binary information encoded in sequence-defined poly(alkoxyamine amide)s. Rapid Commun. Mass Spectrom..

[13] Arnould M.A., Vargas R., Buehner R.W., Wesdemiotis C. (2005). Tandem Mass Spectrometry Characteristics of Polyester Anions and Cations Formed by Electrospray Ionization. Eur. J. Mass Spectrom..

[14] Wang R., Xu S., Wang N., Xia B., Jiang Y., Wang R. (2016). Transcriptome Analysis of Secondary Metabolism Pathway, Transcription Factors, and Transporters in Response to Methyl Jasmonate in Lycoris aurea. Front. Plant Sci..

[15] Ernst M., Bin Kang K., Caraballo-Rodríguez A.M., Nothias L.-F., Wandy J., Chen C., Wang M., Rogers S., Medema M.H., Dorrestein P.C. (2019). MolNetEnhancer: Enhanced Molecular Networks by Integrating Metabolome Mining and Annotation Tools. Metabolites.

[16] Silva D., Ricardo R., Wang M., Nothias L.F., van der Hooft J.J., Caraballo-Roíguez A.M., Fox E., Balunas M.J., Klassen J.L., Lopes N.P. (2018). Propagating annotations of molecular networks using in silico fragmentation. PLoS Comput. Biol..

[17] Nothias L.-F., Petras D., Schmid R., Dührkop K., Rainer J., Sarvepalli A., Protsyuk I., Ernst M., Tsugawa H., Fleischauer M. (2020). Feature-based molecular networking in the GNPS analysis environment. Nat. Methods.

[18] Tang W., Eisenbrand G. (1992). Chinese Drugs of Plant. Origin.

[19] Doi K., Kojima T., Makino M., Kimura Y., Fujimoto Y. (2001). Studies on the Constituents of the Leaves of *Morus alba* L.. Chem. Pharm. Bull..

[20] Yadav M., Tomar R., Prasad G., Jain S., Yadav H. (2008). Complementary Hypoglycemic and Anti-Hyperglycemic Activity of Various Extracts of Fenugreek Seeds in Rats. Asian J. Biochem..

[21] Song W., Wang H., Bucheli P., Zhang P., Wei D., Lu Y. (2009). Phytochemical Profiles of Different Mulberry (*Morus* sp.) Species from China. J. Agric. Food Chem..

[22] Mena P., Sánchez-Salcedo E.M., Tassotti M., Martínez J.J., Hernández F., Del Rio D. (2016). Phytochemical evaluation of eight white (*Morus alba* L.) and black (*Morus nigra* L.) mulberry clones grown in Spain based on UHPLC-ESI-MSn metabolomic profiles. Food Res. Int..

[23] Kim J.Y., Chung H.I., Jung K., Wee J., Kwon O. (2013). Chemical profiles and hypoglycemic activities of mulberry leaf extracts vary with ethanol concentration. Food Sci. Biotechnol..

[24] Park C.H., Park Y.E., Yeo H.J., Yoon J.S., Park S., Kim J.K., Park S.U. (2021). Comparative Analysis of Secondary Metabolites and Metabolic Profiling between Diploid and Tetraploid Morus alba L.. J. Agric. Food Chem..

[25] Nomura T., Fukai T., Katayanagi M. (1977). Kuwanon A, B, C and Oxydihydromorusin, Four New Flavones from the Root Bark of the Cultivated Mulberry Tree (*Morus alba* L.). Chem. Pharm. Bull..

[26] Zheng Z., Zhang Q., Chen R., Yu D. (2012). Four new flavonoids from Morus australis. J. Asian Nat. Prod. Res..

[27] Jung J., Ko W., Park J., Seo K., Oh E., Lee D., Lee D., Kim Y., Lim D., Han D. (2015). Isoprenylated flavonoids from the root bark of Morus alba and their hepatoprotective and neuroprotective activities. Arch. Pharm. Res..

[28] Huang Q., Lei C., Wang P., Li J., Li J., Hou A. (2017). Isoprenylated phenolic compounds with PTP1B inhibition from Morus alba. Fitoterapia.

[29] Ha M.T., Seong S.H., Nguyen T.D., Cho W., Ah K.J., Ma J.Y., Woo M.H., Choi J.S., Min B.S. (2018). Chalcone derivatives from the root bark of Morus alba L. act as inhibitors of PTP1B and α-glucosidase. Phytochemistry.

[30] Waters Corporation (2011). A New Data Dependent Acquisition Alogrithm (FastDDA) for the Rapid Characterization of Complex Mixtures Waters Corporation. Appl. Note.

[31] Pluskal T., Castillo S., Villar-Briones A., Oresic M. (2010). MZmine 2: Modular framework for processing, visualizing, and analyzing mass spectrometry-based molecular profile data. BMC Bioinform..

[32] Shannon P., Markiel A., Ozier O., Baliga N.S., Wang J.T., Ramage D., Amin N., Schwikowski B., Ideker T. (2003). Cytoscape: A Software Environment for Integrated Models of Biomolecular Interaction Networks. Genome Res..

