# Supplementary material for: Investigation of Chemical Profiles of Different Parts of Morus alba Using a Combination of Molecular Networking Methods with Mass Spectral Data from Two Ionization Modes of LC/MS
Source: Plants (Basel). 2021 Aug 19;10(8):1711. doi: 10.3390/plants10081711 (PMC8398940; doi:10.3390/plants10081711)
Supplement: Supplementary file 1 [file plants-10-01711-s001.zip › plants-1329720-supplementary.pptx]

## Slide 1
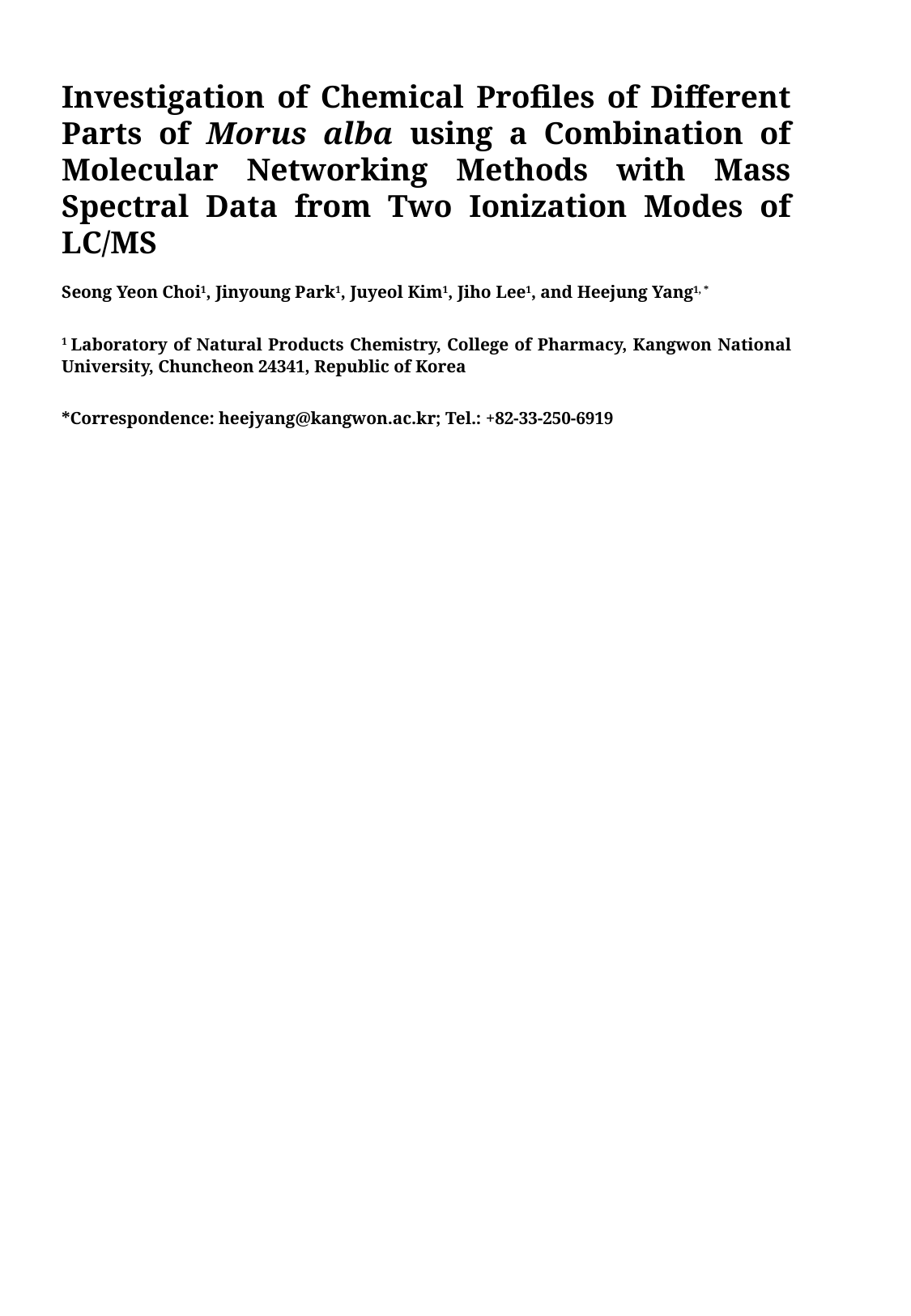

Investigation of Chemical Profiles of Different Parts of Morus alba using a Combination of Molecular Networking Methods with Mass Spectral Data from Two Ionization Modes of LC/MS
Seong Yeon Choi1, Jinyoung Park1, Juyeol Kim1, Jiho Lee1, and Heejung Yang1, *
1 Laboratory of Natural Products Chemistry, College of Pharmacy, Kangwon National University, Chuncheon 24341, Republic of Korea
*Correspondence: heejyang@kangwon.ac.kr; Tel.: +82-33-250-6919

## Slide 2
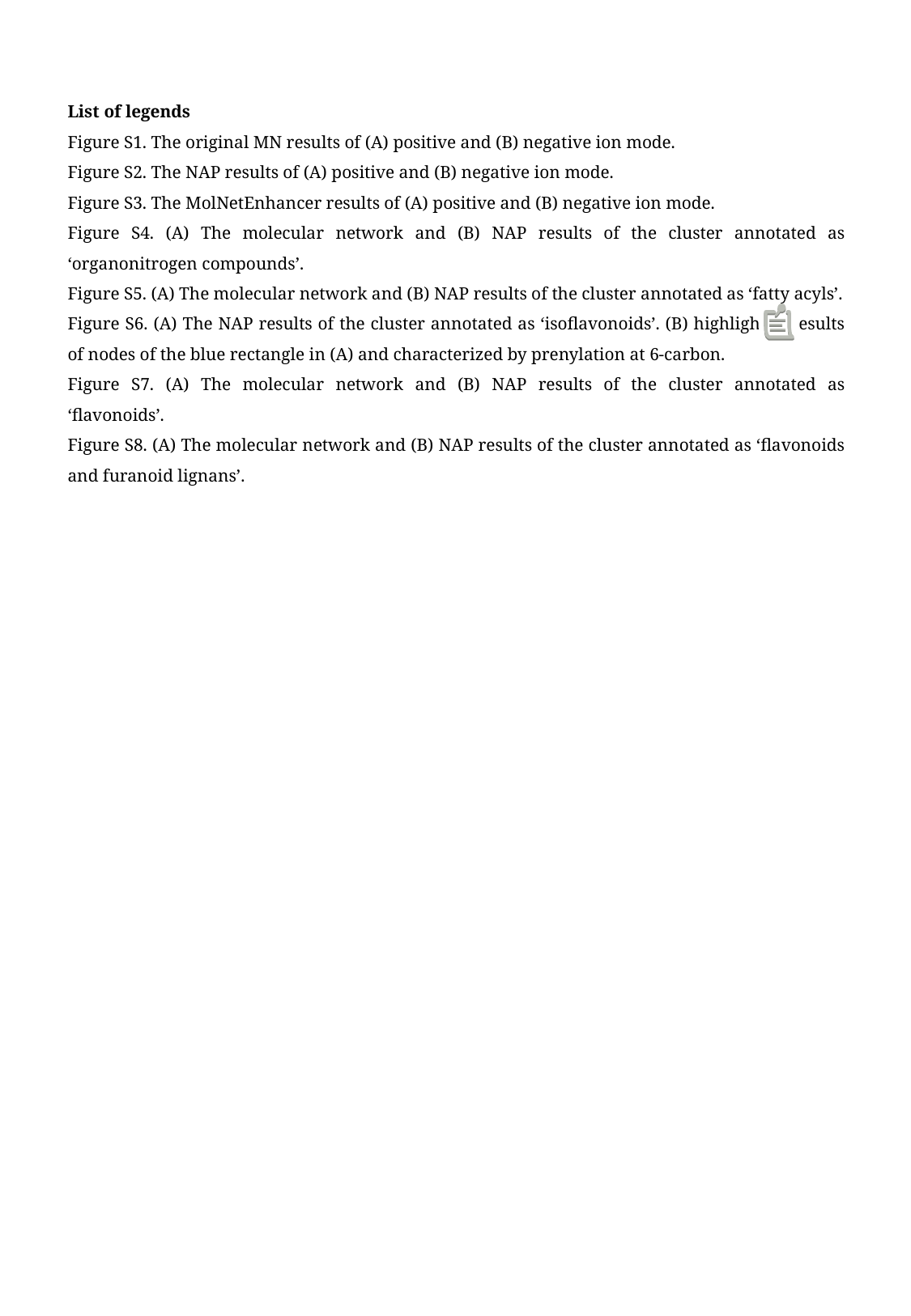

List of legends
Figure S1. The original MN results of (A) positive and (B) negative ion mode.
Figure S2. The NAP results of (A) positive and (B) negative ion mode.
Figure S3. The MolNetEnhancer results of (A) positive and (B) negative ion mode.
Figure S4. (A) The molecular network and (B) NAP results of the cluster annotated as ‘organonitrogen compounds’.
Figure S5. (A) The molecular network and (B) NAP results of the cluster annotated as ‘fatty acyls’.
Figure S6. (A) The NAP results of the cluster annotated as ‘isoflavonoids’. (B) highlighted results of nodes of the blue rectangle in (A) and characterized by prenylation at 6-carbon.
Figure S7. (A) The molecular network and (B) NAP results of the cluster annotated as ‘flavonoids’.
Figure S8. (A) The molecular network and (B) NAP results of the cluster annotated as ‘flavonoids and furanoid lignans’.

## Slide 3
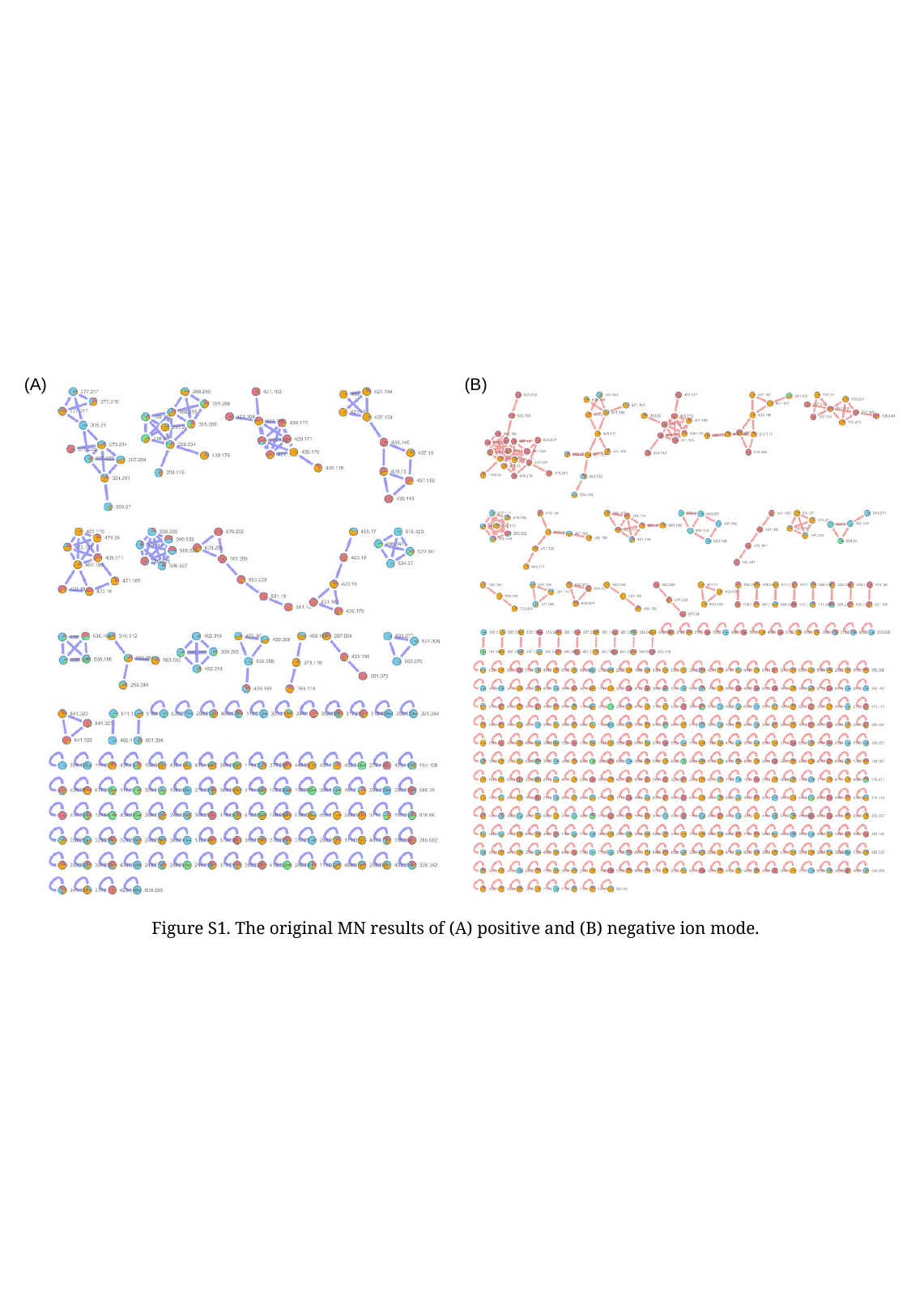

(A)
(B)
Figure S1. The original MN results of (A) positive and (B) negative ion mode.

## Slide 4
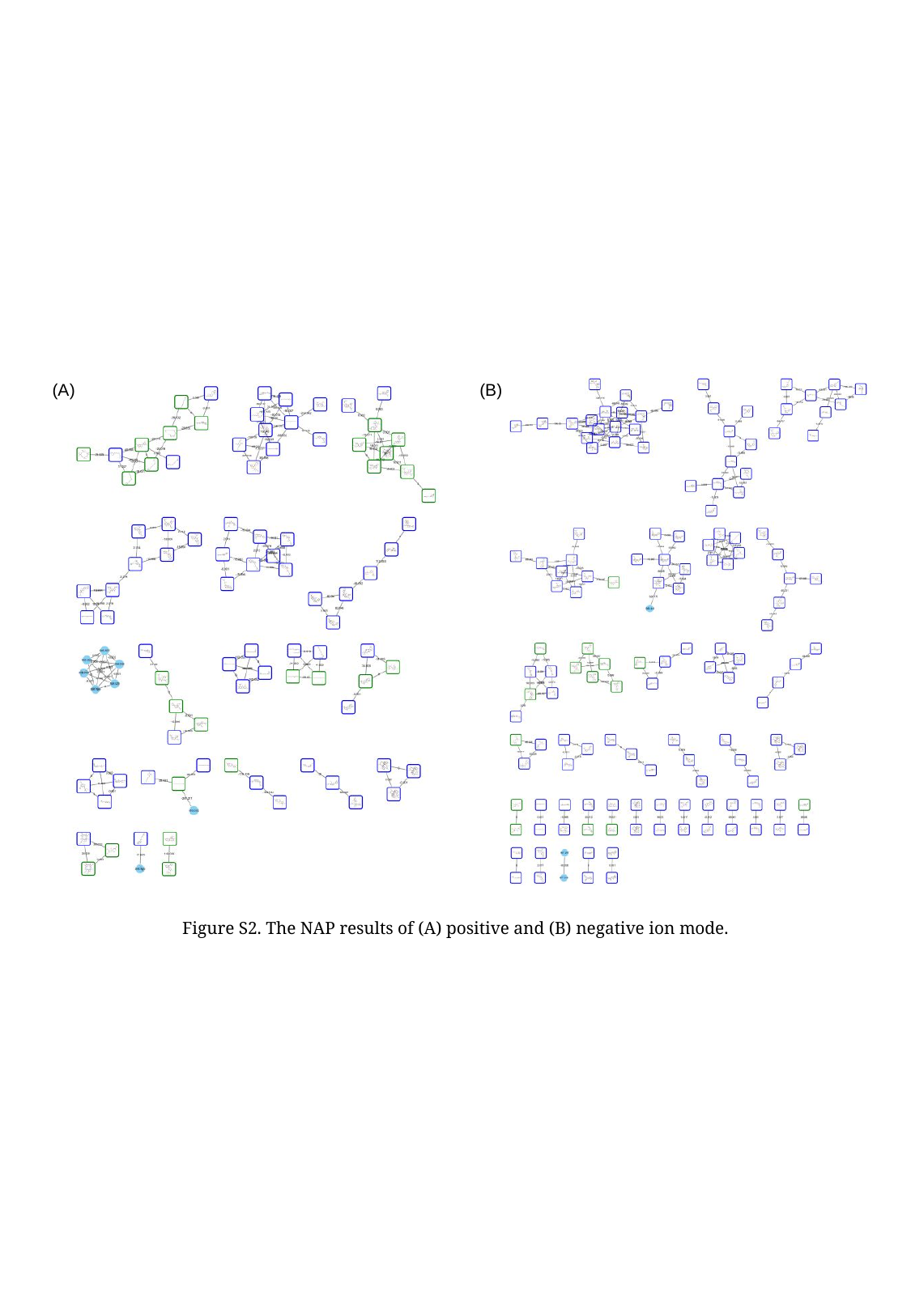

(A)
(B)
Figure S2. The NAP results of (A) positive and (B) negative ion mode.

## Slide 5
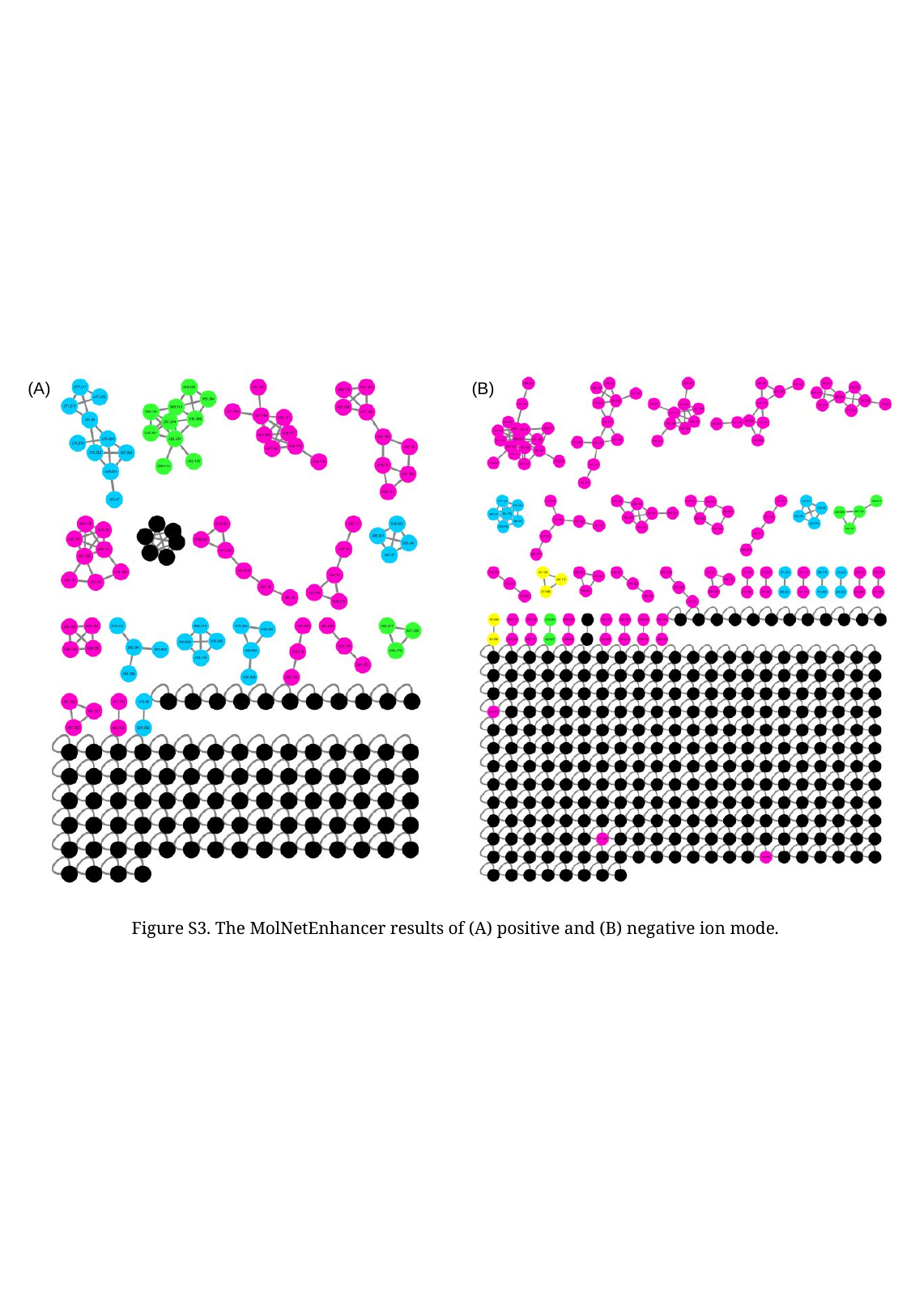

(A)
(B)
Figure S3. The MolNetEnhancer results of (A) positive and (B) negative ion mode.

## Slide 6
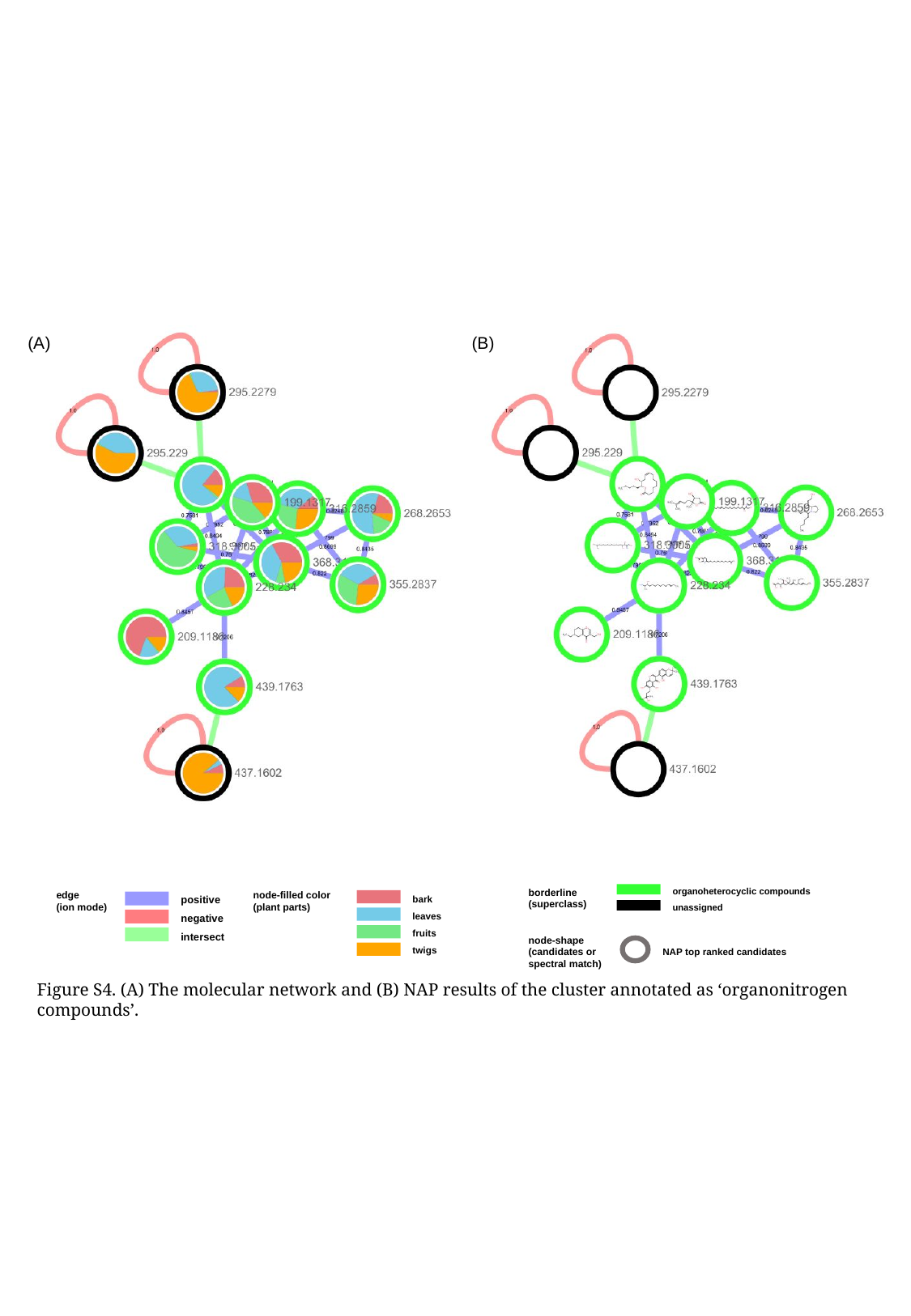

(A)
(B)
organoheterocyclic compounds
unassigned
borderline
(superclass)
node-shape
(candidates or spectral match)
NAP top ranked candidates
positive
negative
intersect
edge
(ion mode)
bark
leaves
fruits
twigs
node-filled color
(plant parts)
Figure S4. (A) The molecular network and (B) NAP results of the cluster annotated as ‘organonitrogen compounds’.

## Slide 7
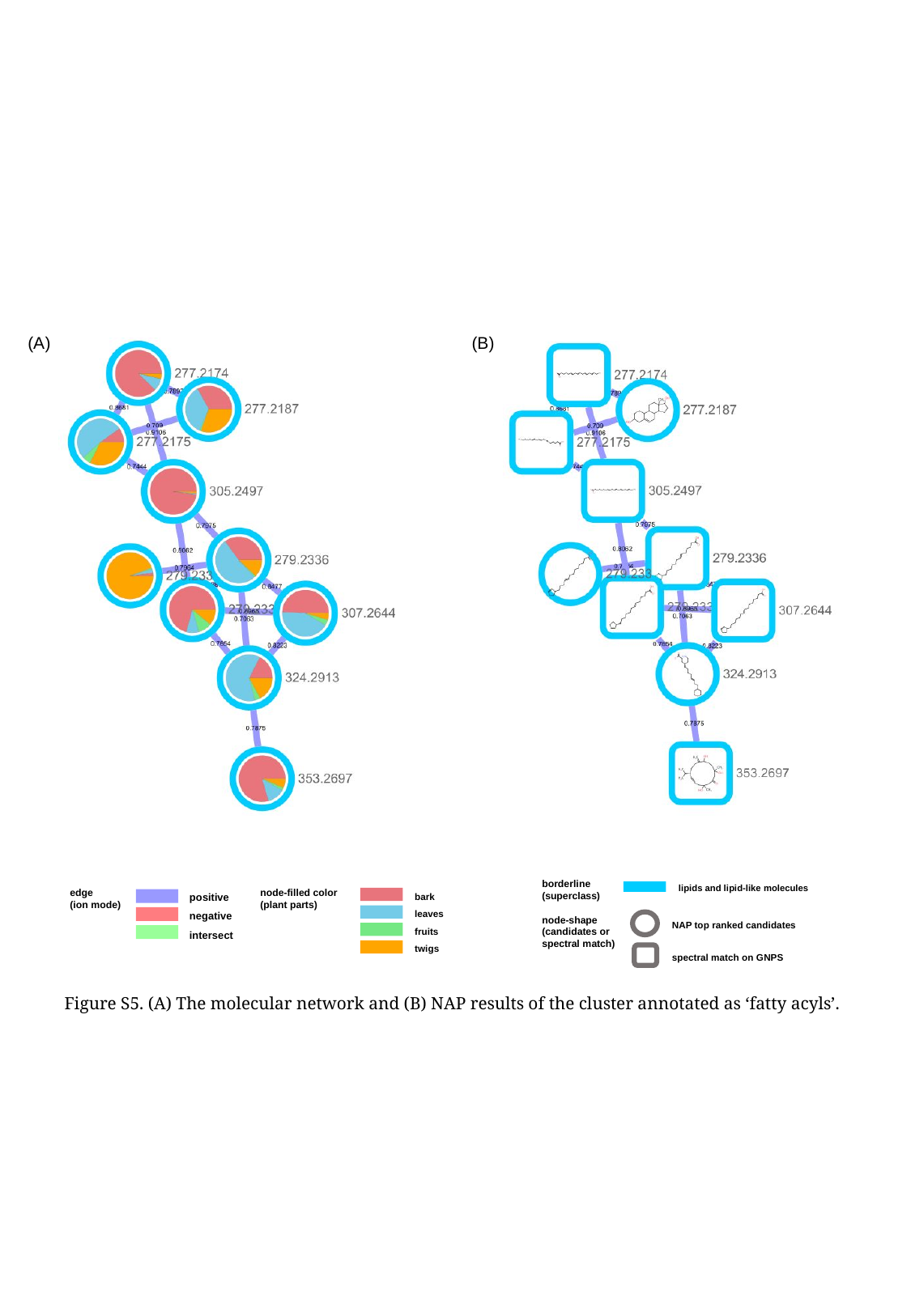

(A)
(B)
lipids and lipid-like molecules
borderline
(superclass)
node-shape
(candidates or spectral match)
NAP top ranked candidates
spectral match on GNPS
positive
negative
intersect
edge
(ion mode)
bark
leaves
fruits
twigs
node-filled color
(plant parts)
Figure S5. (A) The molecular network and (B) NAP results of the cluster annotated as ‘fatty acyls’.

## Slide 8
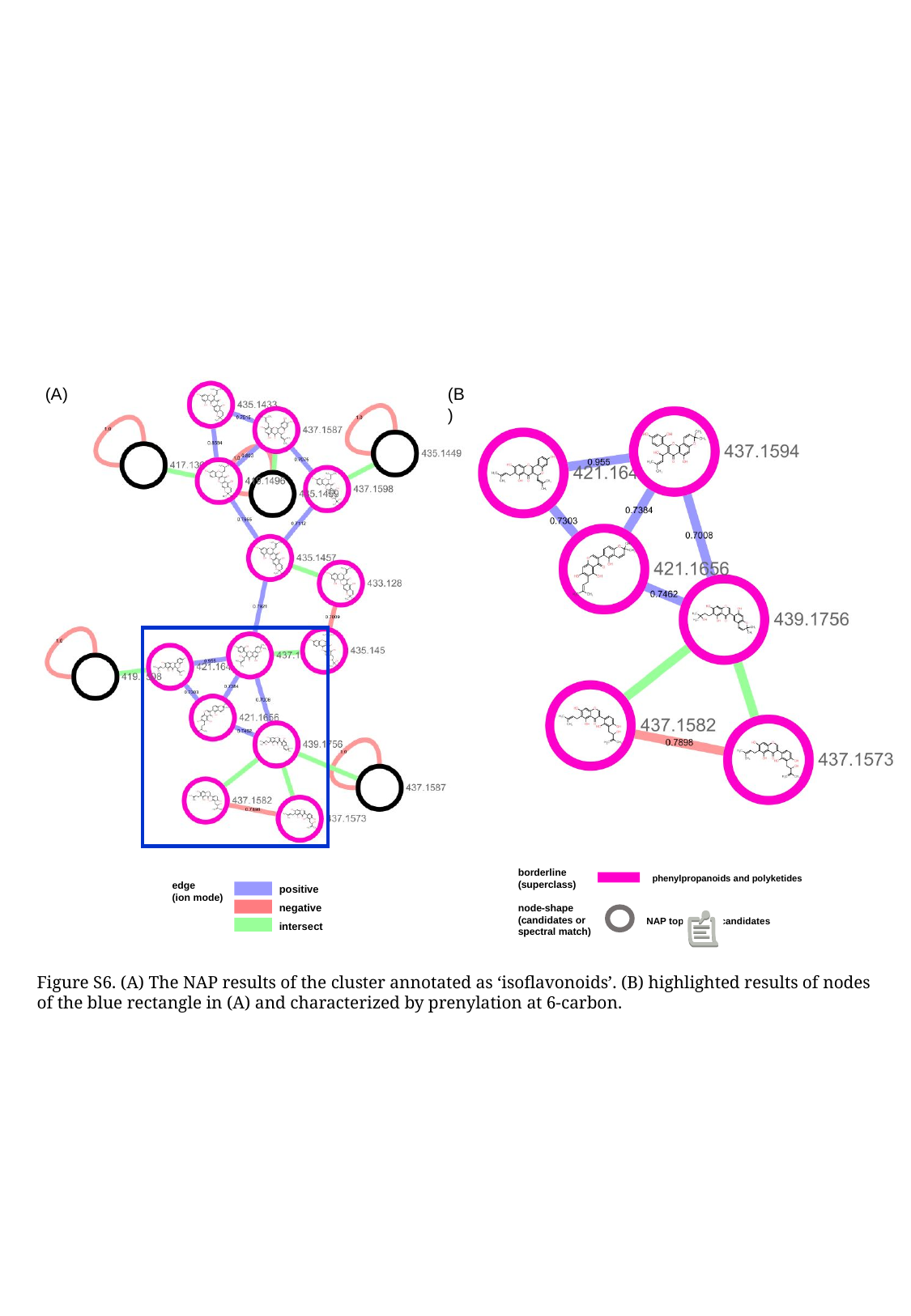

(A)
(B)
borderline
(superclass)
node-shape
(candidates or spectral match)
NAP top ranked candidates
phenylpropanoids and polyketides
positive
negative
intersect
edge
(ion mode)
Figure S6. (A) The NAP results of the cluster annotated as ‘isoflavonoids’. (B) highlighted results of nodes of the blue rectangle in (A) and characterized by prenylation at 6-carbon.

## Slide 9
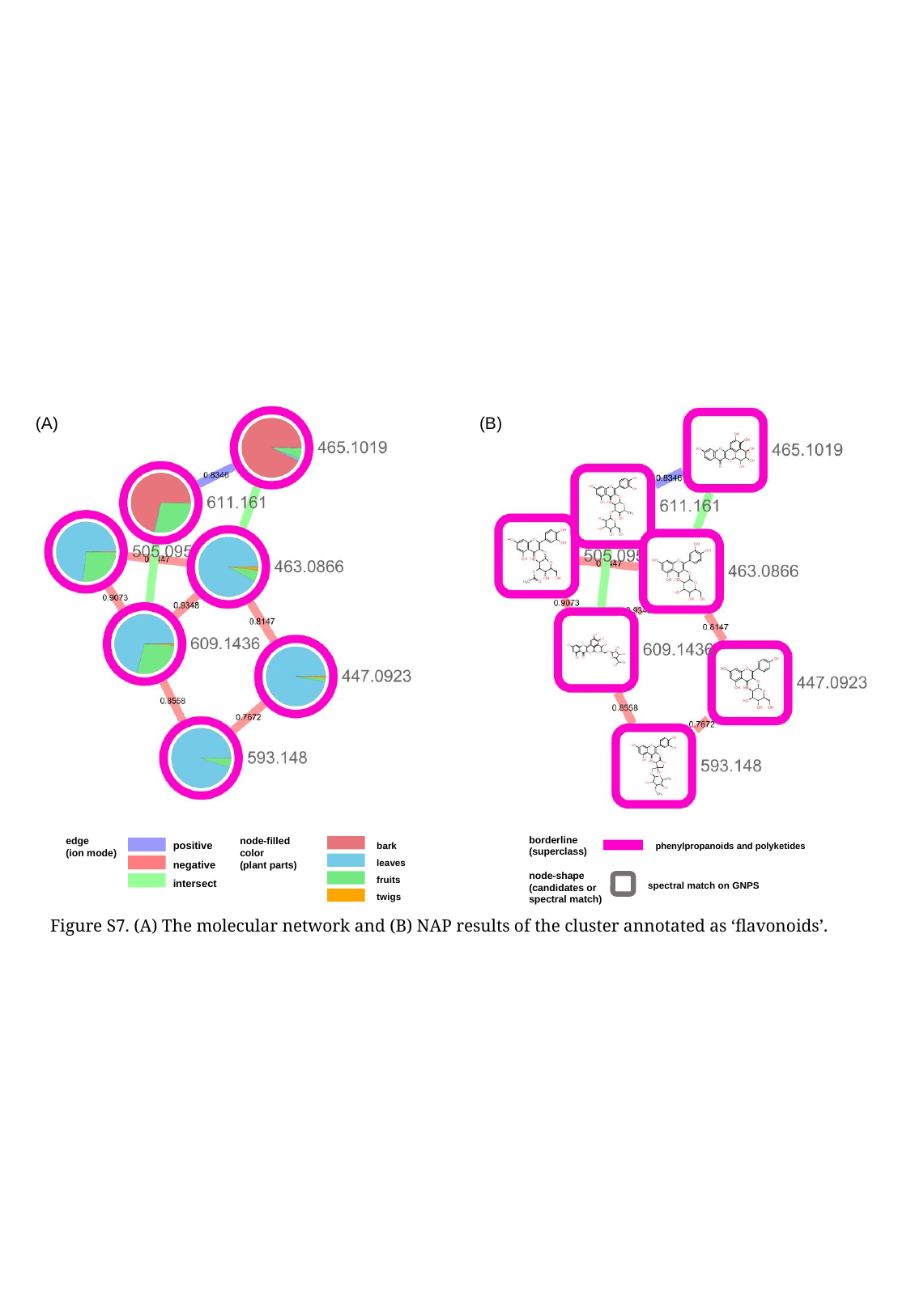

(A)
(B)
positive
negative
intersect
edge
(ion mode)
bark
leaves
fruits
twigs
node-filled color
(plant parts)
borderline
(superclass)
node-shape
(candidates or spectral match)
phenylpropanoids and polyketides
spectral match on GNPS
Figure S7. (A) The molecular network and (B) NAP results of the cluster annotated as ‘flavonoids’.

## Slide 10
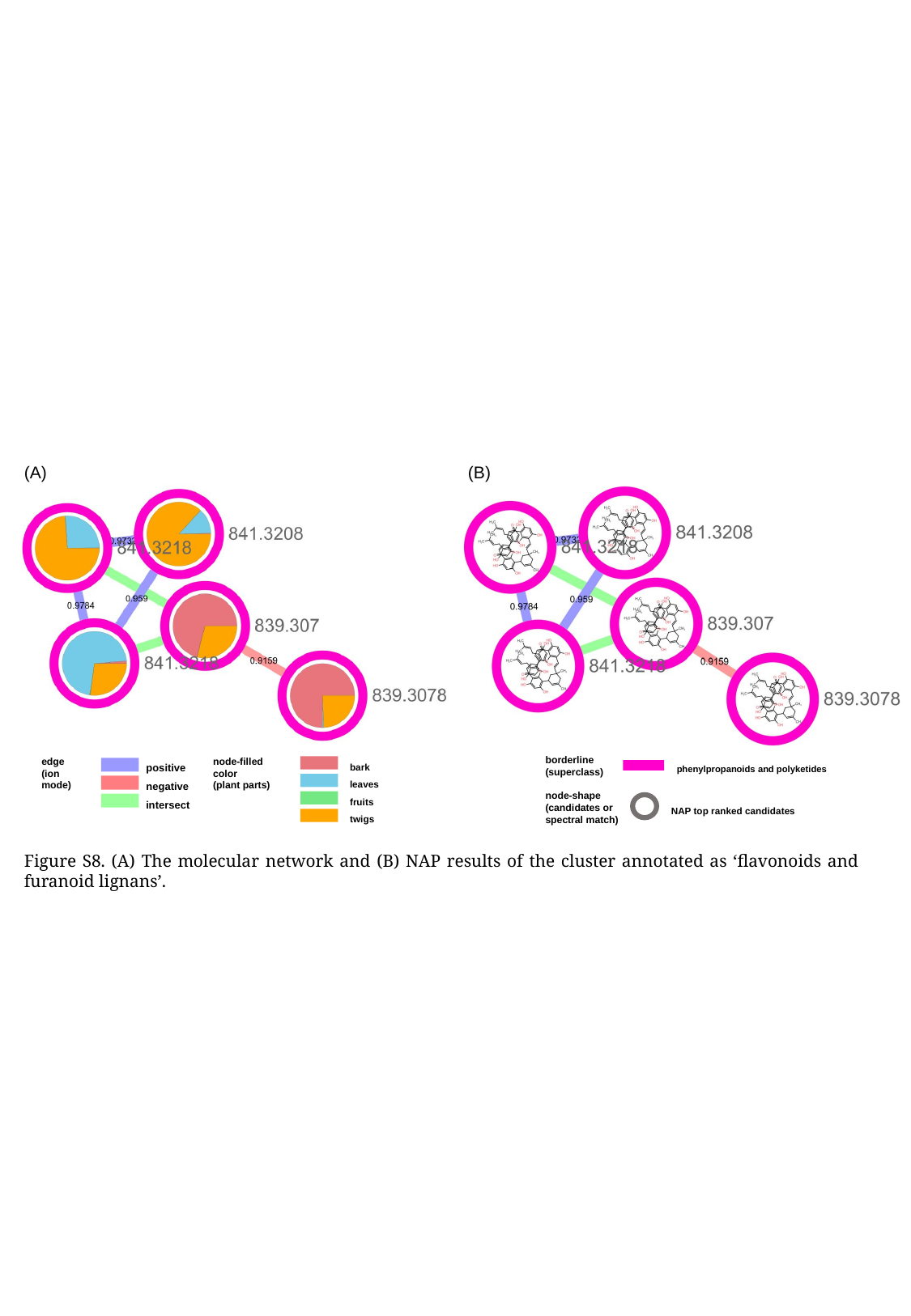

(A)
(B)
borderline
(superclass)
node-shape
(candidates or spectral match)
NAP top ranked candidates
phenylpropanoids and polyketides
edge
(ion mode)
positive
negative
intersect
node-filled color
(plant parts)
bark
leaves
fruits
twigs
Figure S8. (A) The molecular network and (B) NAP results of the cluster annotated as ‘flavonoids and furanoid lignans’.
